# Good clinical and radiological results following remnant-preserving posterior cruciate ligament reconstruction: a systematic review

**DOI:** 10.1007/s00167-022-07192-z

**Published:** 2022-10-08

**Authors:** Riccardo D’Ambrosi, Aurélien Hallé, Alexandre Hardy

**Affiliations:** 1 IRCCS Istituto Ortopedico Galeazzi, Milan, Italy; 2 Dipartimento di Scienze Biomediche per la Salute, Università degli Studi di Milano, Milan, Italy; 3 Service de Chirurgie Orthopedique et Traumatologique, CHU de Cochin, Paris, France; 4 Clinique du Sport, Paris, France

**Keywords:** Posterior cruciate ligament, PCL, Remnant preserving, Knee, Systematic review, Arthroscopy

## Abstract

**Purpose:**

The objective of this systematic literature review was to report the results and complications of recent remnant preservation techniques in posterior cruciate ligament (PCL) reconstruction.

**Methods:**

A systematic review was conducted based on the Preferred Reporting Items for Systematic Reviews and Meta-Analyses (PRISMA) guidelines. Two independent reviewers searched the PubMed, Scopus, Embase, and Cochrane Library databases using the terms “posterior cruciate ligament” or “PCL” and “remnant preserving.” The outcome measures extracted from the studies were the Lysholm score, the International Knee Documentation Committee’s (IKDC) subjective and objective scores, Tegner scores, Orthopädische Arbeitsgruppe Knie (OAK) rate of return to sports, and rate of complications. Data were also extracted from studies that used stress radiographs to perform a quantitative assessment of the preoperative and postoperative anteroposterior stability.

**Results:**

The systematic review included 13 studies. The patient cohort of consisted of 643 participants (544 [84.6%] men and 99 [15.4%] women) with a mean age of 32.9 ± 4.0 years. The mean postoperative follow-up was 34.5 ± 10.9 months (range: 24–96 months), while the mean time from injury to surgery was 14.4 ± 9.9 months (range: 0–240 months). All studies reported clinically significant improvement at final follow-up, as evident from the measured subjective and objective IKDC scores, Lysholm score, Tegner score, and OAK rate. Only three studies reported return to sports activity, with a mean percentage of 90.8% (99/109). All studies showed a significant improvement in posterior translation, from 11.5 ± 1.2 mm to 3.3 ± 1.1 mm, using radiography (side-to-side difference). This systematic review revealed 13 (2.0%) failures and 33 (5.1%) minor complications: 10 (1.6%) cases of stiffness, 21 (4.9%) screws removal, 1 (0.2%) injury of the peroneal nerve, and 1 (0.2%) fibular fracture.

**Conclusions:**

With the currently available data, all studies included in the review on posterior cruciate ligament reconstruction with remnant preservation demonstrated satisfactory outcomes at mid-term follow-up (> 24 months), despite varying surgical techniques and graft types, and intervals from injury to surgery. For clinical relevance, standard PCL reconstruction is a highly effective operation in terms of improvement in functional status, knee stability, quality of life, and cost effectiveness. The remnant preservation technique requires more comprehensive diagnostic assessments of the PCL remnant patterns and more complicated surgical procedures. Given the absence so far of high quality studies with long-term follow-up, the remnant-preserving techniques should be recommended only by experienced knee arthroscopic surgeons.

**Level of evidence:**

Level IV.

**Study registration:**

reviewregistry1376—www.researchregistry.com.

## Introduction

Isolated lesions of the posterior cruciate ligament (PCL) are rare and are linked in most cases to a traffic or sporting accident [18, 35], and they represent only 1% of traumatised knee lesions [21, 26]. PCL is known for its ability to heal properly, and both conservative and surgical treatment provide good functional results for minimal to moderate instability [8, 29]. Nevertheless, long-term studies have shown an increase in the incidence of osteoarthritic lesions and a deterioration in the function of the traumatised knee if treated non-surgically, thus rendering reconstruction of the posterior cruciate ligament more acceptable, given the evolution of surgical techniques [6, 7].

In conventional PCL reconstruction techniques, the remaining fibres of the PCL are generally resected to obtain a good visualisation of the PCL insertion zone and facilitate the passage of the graft [30, 31]. More recently, several researchers have introduced the concept of preservation of the remaining fibres of the PCL; however, the advantages of such preservation are less clear. To our knowledge, there is no recent systematic review on the subject which discusses the interest in performing this technique of preserving PCL fibres. Therefore, the objective of this systematic literature review was to report the results and complications of recent remnant preservation techniques in PCL reconstruction.

It was hypothesised that isolated PCL reconstruction with remnant preservation surgery could provide encouraging clinical outcomes and good recovery of the proprioceptive function.

## Materials and methods

The current systematic review was performed following the Preferred Reporting Items for Systematic Reviews and Meta-Analyses (PRISMA) guidelines and is registered in the Research Registry (reviewregistry1376 – www.researchregistry.com) [24, 28].

### Eligibility criteria

The literature reviewed in this study was selected based on the following criteria.

#### Study design

This review included randomised controlled trials (RCTs) and controlled (non-randomised) clinical trials (CCTs), prospective and retrospective comparative cohort studies, case–control studies, and case series with more than 20 patients and two-year follow-up. Case reports and case series that did not report data on clinical and functional results were excluded.

#### Participants

Studies conducted on skeletally mature patients who underwent PCL reconstruction with the remnant-preserving technique and evaluation through a minimum of two years of follow-up were eligible for the review.

#### Interventions

Studies that reported data on clinical, functional, and radiological outcomes following PCL reconstruction with remnant preservation to treat knee laxity, isolated or associated with other knee ligamentous injuries, were considered eligible for the current study.

#### Types of outcome measures

The outcome measures extracted from the studies were the Lysholm score, the International Knee Documentation Committee’s (IKDC) subjective and objective scores, Tegner scores, the Orthopädische Arbeitsgruppe Knie (OAK) rate of return to sports, and the rate of complications. Data were also extracted from studies that used stress radiographs to perform a quantitative assessment of the preoperative and postoperative anteroposterior stability.

### Information sources and search

A systematic search for relevant literature was performed on the PubMed (MEDLINE), Scopus, EMBASE, and Cochrane Library databases. The publication date was not considered an inclusion criterion. The search was carried out in May 2022. Two independent reviewers (RD and AH) assisted in conducting and validating the search. The following search terms were entered in the title, abstract, and keywords fields: “posterior cruciate ligament” or “PCL” and “remnant preserving.” Only papers published in English were included.

### Data collection and analysis

#### Study selection

The retrieved articles were first screened according to their titles. If an article was found relevant, it was screened further by reading the abstract. After excluding studies that did not meet the eligibility criteria, the entire content of the remaining articles was evaluated for eligibility. To minimise the risk of bias, the authors reviewed and discussed all the selected articles and references, as well as the articles excluded from the study. In the event of any disagreement between the reviewers, the senior investigator made the final decision. At the end of the process, further studies that might have been missed were manually searched by going through the reference lists of the included studies and relevant systematic reviews.

#### Data collection process

The first two authors (RD and AH) collected the data from the selected articles using a computerised tool created with Microsoft Access (Version 2010; Microsoft Corp, Redmond, Washington). Every article was validated again by RD before analysis. For each study, the data collected included information regarding the patients (age, gender, duration between injury and surgery, and follow-up evaluation), their injuries (type, aetiology, and associated injuries), the surgery technique (type of graft used, number of bundles, fixation technique, number of femoral and tibial tunnels, and tensioning protocol), rehabilitation protocol, postoperative outcomes (Lysholm, IKDC, OAK, and Tegner scores, and radiographs), rate of complications, and the rate of return to sports. Rerupture has been defined as repeated PCL revision resulting from trauma [30, 31], graft rupture with persistent subjective instability [6, 7], catastrophic failure [6, 7], or reasons not reported because the revision procedure was performed elsewhere [20]. Scheduled repeated revision resulting from traumatic injury or graft rupture [4] was also considered.

#### Level of evidence

The Oxford Levels of Evidence set by the Oxford Centre for Evidence-Based Medicine were used to categorise the levels of evidence [25].

#### Evaluation of the quality of studies

The quality of the selected studies was evaluated using the Methodological Index for Nonrandomised Studies (MINORS) score [22]. The checklist includes12 items, of which the last four are specific to comparative studies. Each item was given a score of 0–2 points. The ideal score was 16 points for non-comparative studies and 24 for comparative studies.

#### Statistical analysis

The extracted quantitative parameters (age, follow-up time, and results of the PROMs) were given as mean ± standard deviation (SD), when provided in the articles. Otherwise, alternative values like median or range were extracted. Due to the high statistical and methodological heterogeneity of the included studies, a meta-analysis comparing the results between patients with and without concomitant surgeries was not possible. Instead, a narrative description and comparison of the clinical results was performed.

## Results

The electronic search yielded 125 studies. After removing 80 duplicates, 45 studies remained, of which 21 were excluded after reviewing the abstracts, thus bringing down the number of studies eligible for review to 24. An additional 11 articles were excluded based on the inclusion and exclusion criteria.

No additional studies were found upon manually checking the reference lists of the selected articles. Thus, 13 studies were included in the analysis [1, 5, 10, 11, 13–17, 19, 32–34]. Of the 13 included articles, one was level II [32], five were level III [1, 11, 17, 33, 34], and seven were level IV [5, 10, 13–16, 19].

**Fig. 1:**
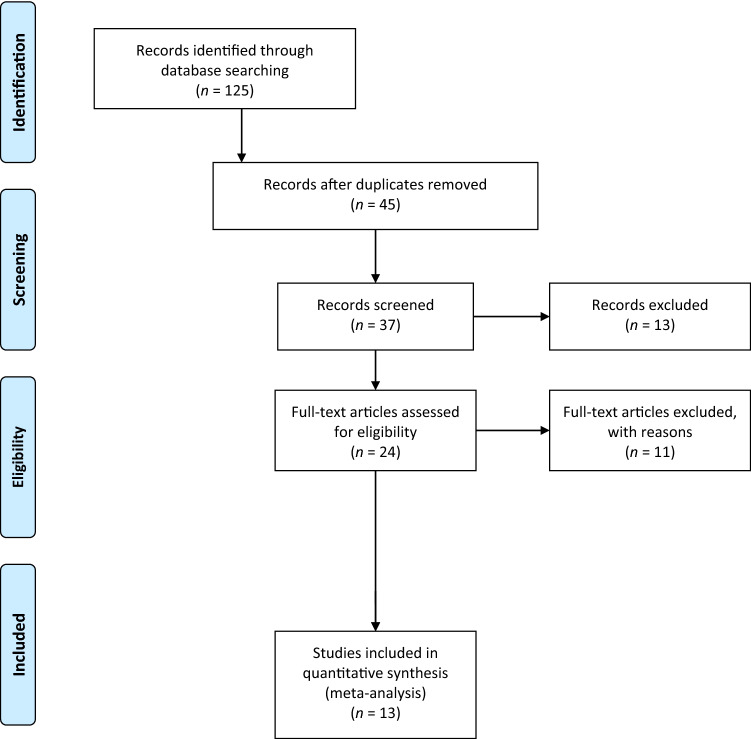
A flowchart of the literature screening performed in this study

Figure 1 is a flowchart depicting the process of selection of the studies. The studies analysed had a mean MINORS score of 13.8 (range: 9–21), which confirmed the acceptable methodological quality of the reviewed literature (Table 1).

**Table 1:** Characteristics of the selected studies

Authors, year	MINORS	Level of evidence	Patients (*n*)	M: F(*n*)	Age mean ± SD (range)	Time between injury and surgery	FU (months)	Aetiology/mechanism of injury (*n*)	Injuries (*n*)
Yoon 2021 [34]	15	III	63 Group A: 33 patients with anatomic femoral tunnel Group B: 30 patients with high femoral tunnel	46:17	Group A: 35.5 ± 15.0 (17–65) Group B: 38.1 ± 17.3 (16–66)	Group A: 18.1 months (0–119) Group B: 10.1 months (0–48)	24	n.a	Group A: 6 chondral injuries and 11 meniscus injuries Group B: 5 chondral injuries and 9 meniscus injuries
Ahn 2005 [1]	15	III	36 Group I: 18 autogenous double-loop hamstring tendon reconstruction Group II: 18 Achilles tendon allograft reconstruction	27:9	Group I: 30 (16–58) Group II: 31 (17–60)	Group I: 9.2 months (5–18) Group II: 8.4 months (4–21)	Group I: 35 (28–55) Group II: 27 (24–36)	15 traffic accidents, 9 sports injuries, 8 accidents during activities of daily living, 4 workrelated injuries	Group I: 5 meniscus injuries Group II: 4 meniscus injuries
Deehan 2003 [5]	9	IV	27	25:2	27 (18–57)	24 months (4–120)	40 months (24–64)	15 direct tackle, 4 patients fell onto the proximal tibia, 2 patients sustained a twisting injury, 2 forced hyperextension of the knee, 1 bull kicked 3 patients motor vehicle accident	6 meniscal injuries 2 medial collateral ligaments lesions
Jung 2010 [10]	12	IV	20	17:3	34.9 (21–49)	1.4 months (9–81 days)	> 24 months	11 proximal peritibial trauma 7 hyperextension 2 unexplained injury	5 posterolateral rotatory instability 4 medial collateral ligament injuries 1 ACL tear
Kim 2012 [11]	19	III	23 without remnant (C group) 30 with remnant (R group)	C: 18:5 R: 27:3	C: 39.4 (14–62) R: 38.0 (17–64)	C: 9 < 6 months 14 > 6 months R: 9 < 6 months 21 > 6 months	C: 48.8 months (24–95) R: 44.7 (24–76)	C: 12 motor vehicle accidents 7 sports injuries 4 accidental fall R: 17 motor vehicle accidents 9 sports injuries 4 accidental fall	All with concomitant posterolateral corner injuries C: 5 meniscal injuries R: 8 meniscal injuries
Lee 2013 [13]	10	IV	20	16:4	36 (17–60)	6 months	61.3 months (31–92)	6 car accidents 6 motorcycle accident 4 sports injury 4 direct injury	3 meniscal tears
Lee 2011 [16]	11	IV	70	62:8	31.2 (16–59)	23.7 (6–240 months)	40.1 months (24–96)	n.a	All with concomitant posterolateral rotatory instability 12 MCL injuries 12 cartilage injuries
Lee 2013 [17]	15	III	74	Group 1 32:2 Group 2 36:4 Group 3 14:1	Group 1 31.1 (18–51) Group 2 31.2 (16–59) Group 3 32.1 (19–24)	n.a	24 months	n.a	n.a
			34 with a PCL remnant-preserving ALB reconstruction (group 1) 40 patients were treated with remnant tensioning and ALB reconstruction (group 2) 15 double-bundle reconstruction if no remnant PCL (group 3)						
Lee 2019 [15]	12	IV	52	47:5	25.8 ± 5.0	2.4 ± 1.9	29.5 ± 8.6	n.a	6 meniscal repair 15 meniscectomy
Lee 2014 [14]	13	IV	92	82:10	35.6	9.4 ± 3.5	48.2 ± 16.2	n.a	47 PLC
Liu 2018 [19]	9	IV	43	36:7	36.6 ± 12.5	4.8 ± 5.7	38.4 ± 8.9	16 car accidents 13 sports injury 10 cycle or motorcycle accident 3 fall 1 hit by weight	7 medial meniscus injuries 4 lateral meniscus 7 cartilage injuries 6 ACL 8 posterolateral corner 8 MCL
Yoon 2011 [32]	21	II	53: 25 single bundle (SB) 28 double bundle (DB)	45:8 SB:20:5 DB: 25:3	SB:28.5 (17–47) DB:27.4 (18–46)	SB: 37 months (3–259) SB: 35 months (3–131)	SB: 31 (24–42) DB: 33 (24–43)	20 Car accident 9 motorcycle accident 12 sports injury 12 falling down	7 medial meniscus injuries 4 lateral meniscus injuries 9 cartilage injuries
Yoon 2021 [33]	19	III	63: 31 anatomic tunnel placement (A) 32: lower tunnel placement (B)	46:17 A: 25:6 B: 21:11	A: 34.0 (17–65) B: 39.4 (16–66)	A: 16.4 months (0–96) B: 12.3 months (0–119)	> 24 months	n.a	11 chondral injuries 20 meniscal injuries

ACL, anterior cruciate ligament; MCL, medial collateral ligament; PLC, posterolateral corner

### Patients’ and study characteristics

**Fig. 2:**
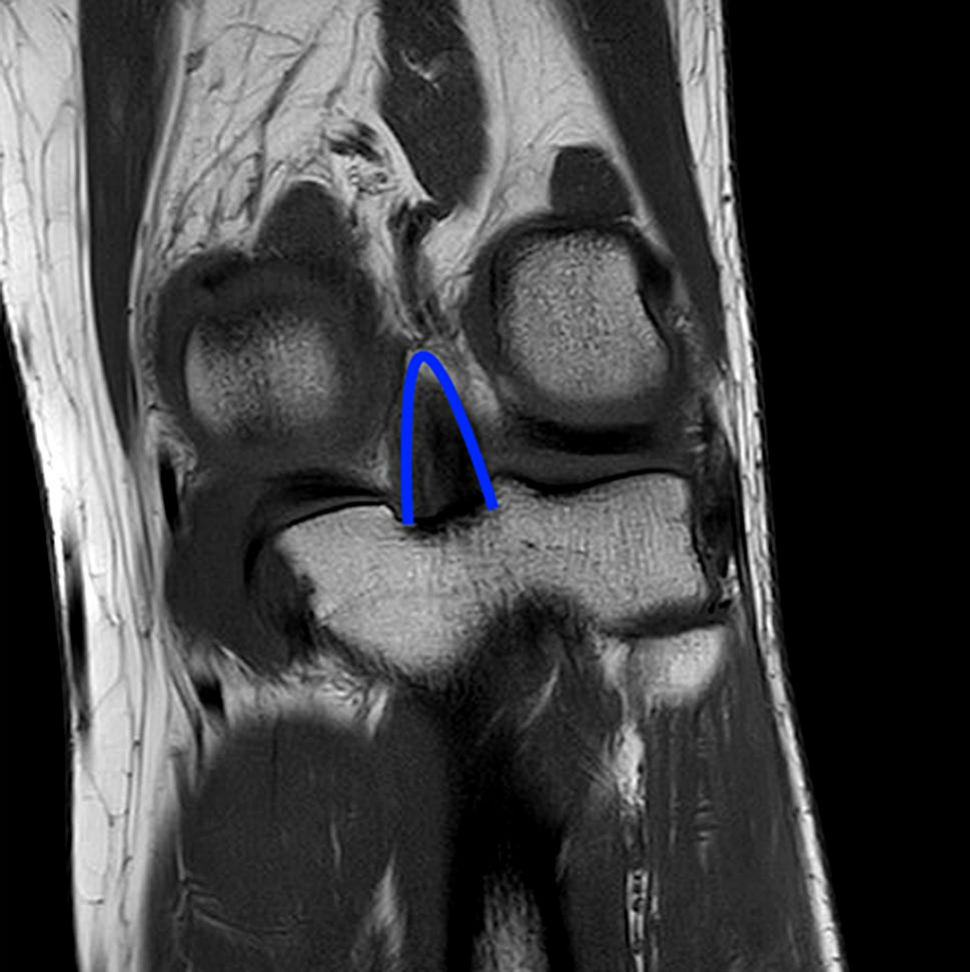
Coronal Magnetic Resonance of the knee showing the remnant of the posterior cruciate ligament (blue lines)

Table 1 shows the characteristics of the cohorts involved in the 13 selected studies, and a summary of their data. The patients cohort consisted of 643 participants (544 [84.6%] men and 99 [15.4%] women) with a mean age of 32.9 ± 4.0 years (range: 14–66). The mean postoperative follow-up was 34.5 ± 10.9 months (range: 24–96 months), while the mean time from injury to surgery was 14.4 ± 9.9 months (range: 0–240 months). Concomitant injuries involved 50 (7.8%) chondral injuries, 109 (17.0%) meniscus injuries, 24 (3.7%) medial collateral ligament (MCL) lesions, 113 (17.6%) posterolateral rotatory instability, and seven (1.1%) ACL tears (Fig. 2).

### Surgical protocol

The data regarding the surgical technique followed in each of the examined studies are displayed in Table 2. The type of graft used in the various studies was as follows: six studies reported the use of Achilles allograft [1, 11, 13, 32–34], five studies autologous hamstrings [1, 5, 10, 16, 17], and three studies tibialis allografts (anterior or posterior) [14, 15, 19]. The femoral graft-fixation construct was performed in 10 studies with a screw [1, 5, 10, 11, 13, 16, 17, 32–34], and with the RigidFix System in three studies [14, 15, 17], while the Endobutton System was employed in one study only [19].

**Table 2:** Surgical and rehabilitation protocol

Lead Author	Graft type	Fixation technique		Surgical technique	Preservation technique	Bundle	Femoral tunnel	Tibial tunnel	Tension protocol	Rehabilitation protocol			
		Femur	Tibia							Brace/splint	Partial WB time	ROM time	Passive and active exercise time
Yoon 2021 [34]	Achilles allograft	CS and spiked washer or staples, as well as bioabsorbable interference	MIS	Transtibial	As much fibre as possible	SB	1	1	90° of flexion	Yes	After 3 weeks	90° at 6th week; 135° at the 3rd month	n.a
Ahn 2005 [1]	Hamstring versus Achilles allograft	CS and washer + BIS	BIS + Post-tie	Outside-in	As much fibre as possible	SB	1	1	n.a	n.a	n.a	n.a	n.a
Deehan 2003 [5]	Hamstring	IS	IS	Anteromedial Portal	Only posteromedial fibres	SB	1	1	60° of flexion	No	Day 0	Day 0	Day 0
Jung 2010 [10]	Hamstring	BIS	BIS	Transtibial	ALL fibres plus synovium	SB	1	1	70° to 90° of flexion	Yes	Day 0	90° by the 6th week 140° by the 12th week	Day 0
Kim 2012 [11]	Achilles allograft	BIS	BIS	Transtibial	As much fibre as possible	SB	1	1	70° of flexion	Yes	Day 0	0° for 4 weeks	Day 0
Lee 2013 [13]	Achilles allograft	BIS	BIS and a cancellous screw and a washer	Anterolateral Portal	As much fibre as possible	SB	1	1	90° of flexion	Yes	4–6 weeks	0° for 3–4 weeks	4–6 weeks
Lee 2011 [16]	Hamstring	BIS	IS + spiked washer	Modified Inlay Technique	ALL bundle	SB	1	1	70° to 90° of flexion	Yes	Day 0	Day 3/5	Hamstring strengthening after 4 months
Lee 2013 [17]	Griup 1: hamstring quadruple Group 2: hamstring quadruple	Group 1: Rigidfix Group 2:BIS	Group 1: IS Group 2:Staple + screw	Group 1: transtibial Group 2: inlay technique	Group 1: ALL bundle Group 2:remnant tensionin + anterolateral bundle reconstruction	Group 1:SB Group 2: SB	Group 1: 1 Group 2: 1	Group 1:1 Group 2:1	Group 1.: 90° Group 2: 70° to 90°	For both groups: Yes	For both groups: Full WB at 8 weeks	For both groups Day 3/5	n.a
Lee 2019 [15]	Tibialis anterior or posterior allograft	RigidFix	2 BIS	Transtibial	As much as possible	SB	1	1	90° of flexion	Yes	Day 0	90° at 4 weeks	3 weeks
Lee 2014 [14]	Tibiali santerior or posterior allograft	RigidFix	2 BIS	Transtibial	As much as possible	SB	1	1	n.a	Yes	Day 0	Full extension for 3 weeks	Day 0
Liu 2018 [19]	Tibialis allograft	Endobutton	Biointrafix	All-anterior approach	As much fibre as possible	SB	1	1	90° of flexion	Yes	7th week	45° at 4 weeks, < 90° from5 to 6 weeks	Day 0
Yoon 2011 [32]	Achilles allograft	SB: cancellous screw and spiked washer or staples DB: BIS	SB/DB: IS	Anteromedial portal	As much fibre as possible	SB/DB	1 or 2	1	90° of flexion	Yes	At 3rd week	90° at 6 weeks	n.a
Yoon 2021 [33]	Achlles allograft	Cancellous screws with a spiked washer	MIS	Anteromedial portal	As much fibre as possible	SB	1	1	90° of flexion	Yes	At 3rd week	90° at 6 weeks	n.a

IS,  interference screw; MIS, metal interference screw; BIS, biobasorbable interference screw; CS, cancellous screw; SB, single bundle; DB, double bundle; ALL, anterolateral

Furthermore, all studies reported conducting the tibial fixation with a screw (with or without adding a staple) [1, 5, 10, 11, 13–17, 32–34], except for one that used the Biointrafix System [19]. Additionally, all studies except one [32] performed a single-bundle reconstruction [1, 5, 10, 11, 13–17, 33, 34]. The tensioning of the PCL reconstruction was performed at 90° in seven studies [13, 15, 17, 19, 32–34], at a range from 70° to 90° in three studies [10, 16, 17], and at 70° and 60° in one study [5, 11]. The use of a postoperative brace was suggested in all studies [1, 10, 11, 13–17, 19, 32–34] except one [5]. Moreover, weight-bearing was allowed from day zero in six studies [5, 10, 11, 14–16], while it was allowed from 3–7 weeks post operation in other studies [13, 17, 19, 32–34].

### Preservation technique

Six studies reported the use of the transtibial technique [10, 11, 14, 15, 17, 34], three studies used the anteromedial portal technique [5, 32, 33], and one study employed the anterolateral portal technique [13]. An outside-in technique was performed in one study [1], and an inlay technique was followed in two studies [16, 17], while an all-anterior approach was used in one study [19]. Different levels of preservation were reported; in this regard, as much fibre of PCL as possible was preserved in nine studies [1, 11, 13–15, 19, 32–34], and in another study, as many fibres as possible and synovium were preserved [10]. Furthermore, only posteromedial fibres were preserved in one study [5], while only anterolateral fibres were preserved in two other studies [16, 17]; on the other hand, a tensioning of the remaining fibres plus anterolateral bundle reconstruction were performed in one study [17].

### Clinical and functional outcomes

**Fig. 3:**
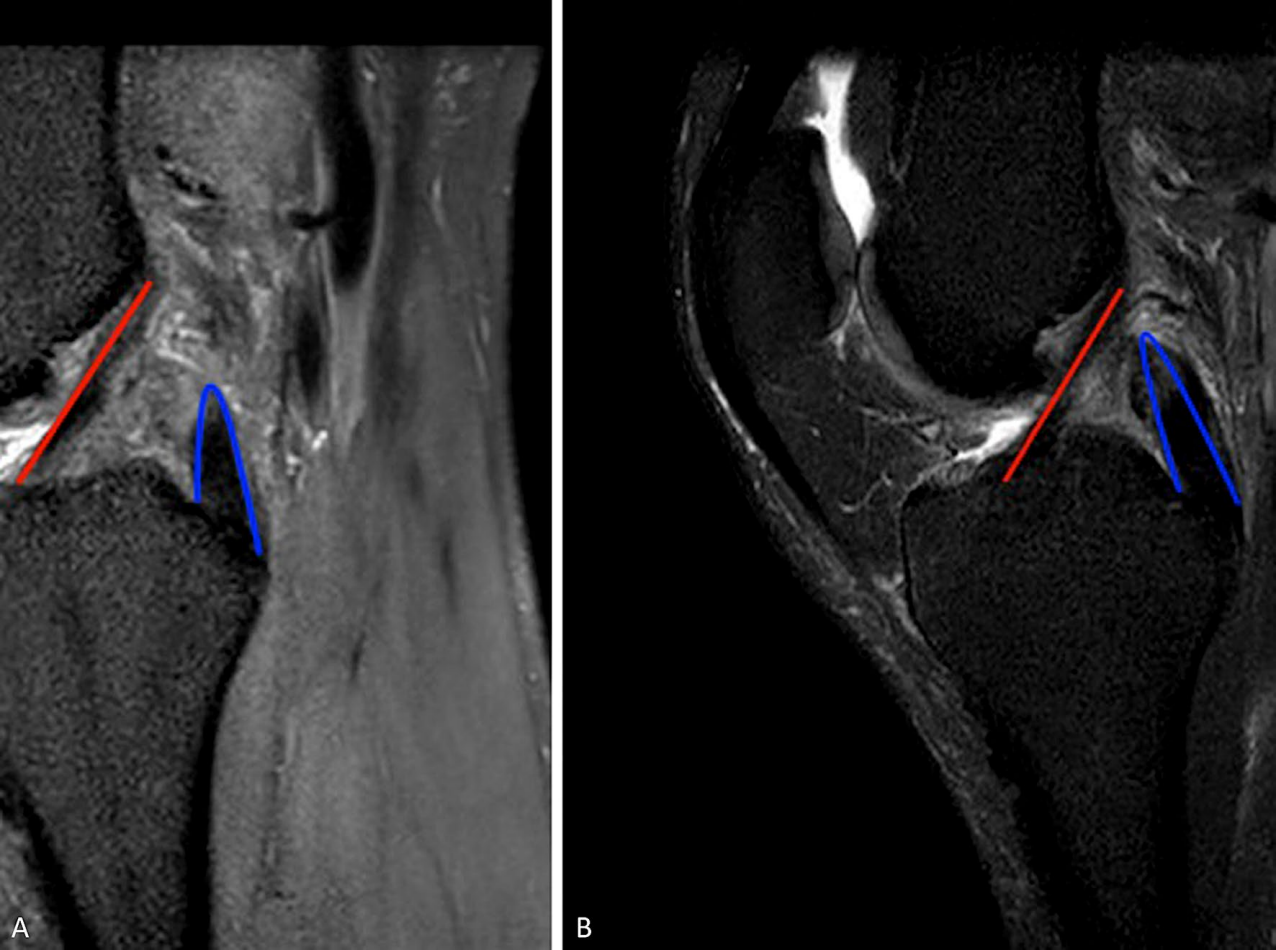
**A**, **B** Sagittal Magnetic Resonance of the knee showing the remnant of the posterior cruciate ligament (blue lines) and the intact anterior cruciate ligament, highlighting difficulties in performing surgery due to limited space

All studies reported clinically significant improvement at final follow-up. The subjective IKDC scores observed in 11 studies [10, 11, 13–17, 19, 32–34] (only at the final follow-up in one study) were measured at 51.2 ± 6.5 at the preoperative stage and 80.2 ± 7.0 at the postoperative stage. On the other hand, eight studies [1, 5, 10, 11, 13, 16, 17, 32] reported the objective IKDC scores (only at the final follow-up in one study); in this regard, at the preoperative stage, the levels were measured as follows: A—0; B—8; C—130; D—143, whereas at the postoperative stage the levels were measured as follows: A—135; B—154; C—35; D—6 (Fig. 3).

Ten studies [1, 5, 11, 13–15, 19, 32–34] evaluated the Lysholm score (only at the final follow-up in one study), with mean results improving from 59.8 ± 4.8 preoperatively to 86.3 ± 5.9 postoperatively. Additionally, seven studies [11, 14, 15, 19, 32–34] (only at the final follow-up in one study) reported the Tegner score varying from 2.9 ± 0.9 preoperatively to 5.6 ± 1.0 postoperatively. Finally, three studies [10, 16, 17] reported OAK scores, with the mean results improving from 65.0 ± 3.5 at the preoperative stage to 88.0 ± 1.6 at the postoperative stage. It is noteworthy that only three studies [5, 11, 15] reported a return to sports activity, with a mean percentage of 90.8% (99/109). In particular, in the study by Deehan et al. the level of activity was evaluated as moderate to strenuous [5]; Kim reported that only eight patients returned to pre-injury activity, while the others were considered near-returns to activity [11], whereas in Lee’s study all patients returned to their pre-injury activity [15].

The results are presented in detail in Table 3.

**Table 3:** Clinical and functional outcomes, complications, and return to sports and activity

	Subjective IKDC		Objective IKDC		Lysholm Score		Tegner		OAK		Radiographic Changing		Complications	Failures	Return to sport
	Pre	Post	Pre	Post	Pre	Post	Pre	Post	Pre	Post	Pre	Post			
Yoon 2021 [34]		Group A: 72.5 ± 11.5 (47.1–90.8) Group B: 75.5 ± 11.8 (48.3–96.6)				Group A: 77.3 ± 12.6 (41.0–100.0) Group B: 77.0 ± 13.1 (45.0–99.0)		Group A: 5.0 (3.0–9.0) Group B: 6.0 (3.0–9.0)				Osteoarthritis Progression: Group A: 5 Group B: 4 Posterior Translation: Group A: 5.2 ± 2.9 mm (0.0–13.0) Group B: 5.2 ± 2.7 mm (0.0–12.5)		Group A: 4 Group B: 1	
Ahn 2005 [1]			Group I: A 0 B 0 C 7 D 11 Group II: A 0 B 0 C 8 D 10	Group I: A 7 B 9 C 2 D 0 Group II: A 2 B 12 C 3 D 1	68 (54–79) Group I: 68.2 (54–78) Group II: 68.6 (54–79)	89 (70–100)* Group I: 90.1* Group II: 85.8*					Posterior Translation: 14 mm (10–20) Group I: 14.3 mm (10–20) Group II: 13.8 mm (10–21)	Posterior Translation: 2.6 mm (0–7)* Group I: 2.2 mm (0–7)* Group II: 2.9 mm (1–7)*	1 stiffness 19 screws removal	0	
Deehan 2003 [5]			A 0 B 7 C 23 D 1	A 15 B 10 C 1 D 1	64 (51–66)	94 (84–94)*							1 stiffness 2 screw removal	1	17 patients participating at this level of activity on a regular basis
Jung 2010 [10]	45.8 ± 15.6	85.4 ± 10.2*	A 0 B 1 C 2 D 17	A 7 B 10 C 2 D 1					61.6 ± 13.1	88.2 ± 9.5*	Posterior translation: 10.4 mm ± 2.1	Posterior translation: 3.0 mm ± 2.6*		1	
Kim 2012 [11]	41.6 ± 10.5	70.6 ± 7.9*	A 0 B 0 C 12 D 18	A 7 B 18 C 4 D 1	60.4 ± 8.9	84.1 ± 10.7*	2.4 (1–4)	4.3 (2–7)*			Posterior Translation: 12.8 mm ± 4.6	Posterior Translation: 4.1 mm ± 3.4*			8 return to activity 22 near-return to activity
Lee 2013 [13]	62.72 ± 10.53	85.41 ± 7.97*	A 0 B 0 C 12 D 8	A 8 B 12 C 0 D 0	70.0 ± 6.89	88.9 ± 4.36*					Posterior Translation: 10.8 mm ± 1.5	Posterior Translation: 3.2 mm ± 1.36*			
Lee 2011 [16]	50.6 ± 16.8	79.7 ± 13.3*	A 0 B 0 C 42 D 28	A 30 B 34 C 6 D 0					63.5 ± 10.4	88.9 ± 7.6*	Posterior translation: 10.3 mm ± 2.4	Posterior translation: 2.2 ± 1.5*	1 injury of the peroneal nerve 1 fibular fracture	1	
Lee 2013 [17]	Group 1: 46.7 ± 16.6 Group 2: 63.5 ± 10.4	Group 1: 65.1 ± 18.4* Group 2: 79.7 ± 13.3*	Group 1: A 0 B 0 C 11 D 23 Group 2: A 0 B 0 C 13 D 27	Group 1: A 21 B 9 C 3 D 1 Group 2: A 17 B 19 C 4 D 0					Group 1: 71.7 ± 9.3 Group 2: 63.5 ± 10.4	Group 1: 85.0 ± 6.7 Group 2: 88.9 ± 7.6_*	Posterior Translation: Group 1: 10.1 ± 2.5 mm Group 2: 10.6 ± 2.4 mm	Posterior Translation: Group 1: 2.3 ± 1.4 mm* Group 2: 2.3 ± 1.5 mm			
Lee 2019 [15]	51.8 ± 10.7	88.7 ± 14.1			54.9 ± 11.2	89.4 ± 12.3*	Pre injury 7.9 ± 1.4 pre-surgery: 4.3 ± 1.5	7.8 ± 1.4*			Posterior Translation: 12.6 ± 2.7 mm	Posterior Translation: 2.9 ± 1.4 mm			100% of the patients,
Lee 2014 [14]	53.3 ± 9.6	86.2 ± 6.1*			56.7 ± 7.1	89.3 ± 7.3*	2.5 ± 0.8	5.1 ± 1.3*			Posterior Translation: 12.1 ± 2.5 mm	Posterior Translation: 2.7 ± 1.3 mm*			
Liu 2018 [19]	51.4 ± 8.9	89.9 ± 6.1*			53.8 ± 11.0	91.9 ± 7.2*	1.9 ± 1.4	5.2 ± 1.2*				MRI: Gross Appearance – Grade 1: 29 Grade II: 14 Grade III: 0 Mean signal intensity score: 1.5 ± 2.8			
Yoon 2011 [32]	SB:40.2 (27.6–46.0) DB: 39.1 (27.6–48.3)	SB: 79.3 (59.8–88.5)* DB: 81.7 (65.5–88.5)*		SB: A 6 B 12 C 6 D 1 DB: A 15 B 9 C 4 D 0	SB: 64 (41–73) DB: 62 (43–71)	SB: 89 (71–99)* DB: 91 (76–100)*	SB: 2 (1–3) DB:2 (1–3)	SB: 6 (4–7)* DB: 6 (4–7)*					3 stiffness		
Yoon 2021 [33]	Group A: 57.4 (16.1–86.2) Group B: 53.0 (19.5–87.4)	Group A: 75.4 (47.1–94.3)* Group B: 72.5 (47.1 -96.6)*			Group A: 60.2 (11.0–95.0) Group B: 56.6 (24.0–90.0)	Group A: 79.2 (45.0–98.0)* Group B: 75.1 (41.0–100)*	Group A: 4.4 (2.0–8.0) DB:3.6 (1.0–6.0)	Group A: 5.5 (3.0–9.0)* Group B: 5.2 (3.0–8.0)*			Posterior Translation: Group A:10.7 mm (5.3–16.1) Group B: 10.4 mm (4..9–15.9)	Posterior Translation: Group A: 5.2 mm (0–13)* Group B: 5.1 mm (0–11.5)*	5 stiffness (3 group A and 2 group B)	2 Group A 3 Group B	

^*^Statistical significant improvement

^§^Only remnant group has been reported in table

*IKDC* International Knee Documentation Committee; *OAK* Orthopädische Arbeitsgruppe Knie; *MRI* magnetic resonance imaging

### Radiological outcomes

Eleven studies reported radiographic evidence of significant improvement at the final follow-up, regarding posterior translation [1, 10, 11, 13–17, 19, 33, 34]. In particular, the mean results of posterior translation improved from 11.5 ± 1.2 mm preoperatively to 3.3 ± 1.1 mm postoperatively. Eight studies reported comparative results, with regard to the contralateral side' [10, 13–17, 33, 34].

Moreover, nine (1.4%) patients reported osteoarthritis progression, while the gross appearance in MRI findings demonstrated grade I osteoarthritis in 29 patients (4.5%) and grade II in 14 patients (2.2%), with a mean signal intensity of 1.5. The results are detailed in Table 3.

### Complications and failures

This systematic review revealed 13 (2.0%) failures and 33 minor complications (5.1%), including 10 cases of stiffness (1.6%), 21 (4.9%) screw removals, 1 (0.2%) injury of the peroneal nerve, and 1 (0.2%) fibular fracture.

The results are reported in detail in Table 3.

## Discussion

The most important findings of the current study confirm that preservation of the remnants of the PCL reconstruction leads to good clinical results, regardless of the technique used. Regarding the clinical results, three types of grafts were used in the different studies, none of which stood out as having a clear benefit. Ahn et al. [1] arrived at the same conclusions when comparing the clinical results of arthroscopic PCL reconstruction using hamstrings or Achilles tendon allograft while preserving the remnant. Lee et al. [15] demonstrated that arthroscopic PCLR with remnant preservation had high return-to-sport rates and a high level of patient satisfaction. The average IKDC was 88.7 ± 14.1, and the average Lysholm score was 89.4 ± 12.3, with a minimum follow-up of two years.

Unlike for the anterior cruciate ligament (ACL), the extant literature on the posterior cruciate ligament (PCL) does not demonstrate the superiority of these techniques compared to conventional techniques [9, 12, 23], due to a lack of prospective, randomised studies, making it hard to decide whether preserving the remnant is advantageous or not. Nevertheless, some authors have described favourable results (IKDC rating of A or B in 97% to 100%) after chronic PCLR (> 6 months) ([1]. While these results are satisfactory, they are still not superior to those obtained using conventional techniques without preservation of the PCL stump, as shown by Kim et al. [11], with similar Lysholm scores, return-to-activity rate, and objective IKDC scores.

Similarly, regarding radiological results, Kim et al. [11] found satisfactory but comparable long-term radiological results. There is no statistically significant difference in the results on long-term stability between patients who underwent PCLR with or without remnant preservation: the mean differences in posterior tibial translation were similar in the two groups (classic group: 4.4 ± 3.0 mm; 82.6 ± 11.0; A: 21.7% and B: 73.9%; and preservation group: 4.1 ± 3.4 mm; 84.1 ± 10.7; A: 26.7% and B: 83.3%; *p* = …/n.s.).

On the other hand, these techniques do not seem to be associated with high complication rates; therefore, they appear feasible without too much difficulty. In this regard, this systematic literature review found only 13 (3%) failures and 10 cases of stiffness (2.35%), which is comparable with other techniques [2, 3, 27].

Some doubts still exist regarding the time from injury to surgery and how it can affect graft maturation in remnant preservation; correlation analysis showed that the time from injury to surgery had a statistically significant correlation with the signal intensity score [19]. Multivariate stepwise logistic regression analyses also showed that time from injury to surgery was the significant covariate [19]. These results imply that the time from injury to surgery might be a risk factor for graft signal intensity. However, the correlations were weak. Similarly, Lee et al. found that chronic groups were significantly inferior to acute or subacute groups in terms of side-to-side laxity difference at follow-up and observed no or very weak PCL remnant in the patients of subacute or chronic groups [17].

This systematic literature review has several limitations. First, the selection of articles was very restrictive as only studies with a minimum follow-up of two years and at least two cases were retained. Moreover, this literature review was based on low quality studies: level II, III and IV studies were included in the analyses, but no level I study was found. These factors may have affected the study’s conclusions. Another limitation is that there was only a direct comparison between remnant-preserving PCLRs and standard PCLRs, although conclusions could be drawn from such a comparison. Furthermore, most of the articles focussed on a surgical technique and reported retrospective series criteria. Additionally, the included studies demonstrated some heterogeneity regarding the type of reconstruction, the type of graft, the type of fixation, and the interval between injury and surgical management. Despite these discrepancies, all studies on PCLR with remnant preservation have reported satisfactory results. Accordingly, this opens up the possibility of new randomised studies concerning the type of reconstruction with preservation of the remnant.

For clinical relevance, standard PCL reconstruction is a highly effective operation in terms of improvement in functional status, knee stability, quality of life, and cost effectiveness. The remnant preservation technique requires more comprehensive diagnostic assessments of the PCL remnant patterns and more complicated surgical procedures. Given the current absence of high quality studies with long-term follow-up, the remnant-preserving techniques should be recommended only by experienced knee arthroscopic surgeons.

## Conclusions

With the currently limited available data, all studies included in the review on PCLR with remnant preservation demonstrated satisfactory outcomes at mid-term follow-up despite their using different surgical techniques and different graft types, and the varying intervals from injury to surgery. However, the review did not find substantial evidence to support the superiority of the new techniques over the traditional ones.
